# Enhancing the Anticancer Activity of Sorafenib through Its Combination with a Nitric Oxide Photodelivering β-Cyclodextrin Polymer

**DOI:** 10.3390/molecules27061918

**Published:** 2022-03-16

**Authors:** Francesca Laneri, Adriana C. E. Graziano, Mimimorena Seggio, Aurore Fraix, Milo Malanga, Szabolcs Béni, Giuseppe Longobardi, Claudia Conte, Fabiana Quaglia, Salvatore Sortino

**Affiliations:** 1 PhotoChemLab, Department of Drug and Health Sciences, University of Catania, I-95125 Cayania, Italy; francesca.laneri@phd.unict.it (F.L.); acegraz@unict.it (A.C.E.G.); mimiseggio@gmail.com (M.S.); fraix@unict.it (A.F.); 2 CycloLab, Cyclodextrin R&D Ltd., Illatos út 7, H-1097 Budapest, Hungary; milomalanga@gmail.com; 3 Department of Pharmacognosy, Semmelweis University, Üllői út 26, H-1085 Budapest, Hungary; beni.szabolcs@pharma.semmelweis-univ.hu; 4 Department of Pharmacy, Drug Delivery Laboratory, University of Napoli Federico II, I-80131 Napoli, Italy; giuseppe.longobardi@unina.it (G.L.); claudia.conte@unina.it (C.C.)

**Keywords:** chemotherapy, light, nitric oxide, sorafenib, cyclodextrin polymers

## Abstract

In this contribution, we report a strategy to enhance the therapeutic action of the chemotherapeutic Sorafenib (SRB) through its combination with a multifunctional β-cyclodextrin-based polymer able to deliver nitric oxide (NO) and emit green fluorescence upon visible light excitation (PolyCDNO). The basically water-insoluble SRB is effectively encapsulated in the polymeric host (1 mg mL^−1^) up to a concentration of 18 μg mL^−1^. The resulting host-guest supramolecular complex is able to release SRB in sink conditions and to preserve very well the photophysical and photochemical properties of the free PolyCDNO, as demonstrated by the similar values of the NO release and fluorescence emission quantum efficiencies found. The complex PolyCDNO/SRB internalizes in HEP-G2 hepatocarcinoma, MCF-7 breast cancer and ACHN kidney adenocarcinoma cells, localizing in all cases mainly at the cytoplasmic level. Biological experiments have been performed at SRB concentrations below the IC_50_ and with light doses producing NO at nontoxic concentrations. The results demonstrate exceptional mortality levels for PolyCDNO/SRB upon visible light irradiation in all the different cell lines tested, indicating a clear synergistic action between the chemotherapeutic drug and the NO. These findings can open up exciting avenues to potentiate the anticancer action of SRB and, in principle, to reduce its side effects through its use at low dosages when in combination with the photo-regulated release of NO.

## 1. Introduction

Sorafenib (SRB) is a multi-kinase inhibitor, approved by the U.S. Food and Drug Administration, currently used in patients for the cure of hepatocellular and advanced renal cell carcinomas [1,2]. Preclinical studies have also shown that SRB exhibits effective antitumor activity in various tumor cells, including breast cancer [3], melanoma [4], thyroid [5] and colorectal carcinomas [6] cell lines. An abnormal increase of tiredness, gastrointestinal disturbance and rashes are the most common side effects associated to the use of this drug that make dose reduction and temporary discontinuation necessary [7]. SRB belongs to the BCS II class of drugs with low aqueous solubility under physiological conditions (ca. 10 ng mL^−1^) [8] and fast metabolism, i.e., the bioavailability of SRB is very low. For these reasons, a number of nanocarriers have been proposed over the last years to improve its therapeutic profile [9]. Of these, polymeric-based nanocarriers have shown great potential for the systemic treatment of liver tumors and fibrosis [10,11,12,13,14,15].

However, SRB resistance in hepatocellular carcinoma HCC remains one of the most significant obstacles in the achievement of efficient chemotherapy [16]. Combination chemotherapy represents nowadays a cornerstone to overcome resistance [17]. This treatment modality aims to combine multiple therapeutic agents to enhance the therapeutic outcome through synergistic or additive effects. At this regard, the use of nitric oxide (NO) in combination with chemotherapeutics is emerging as an intriguing strategy to overcome multidrug resistance (MDR) and to potentiate the overall antitumor activity [18,19]. NO is a small, inorganic free radical that, besides being a well-known bioregulator of vital functions in the human body, plays a key role in tumor biology [20]. NO can exert anticancer action directly, as a cytotoxic agent [21], or indirectly, inhibiting the efflux pumps mainly responsible for MDR, reducing the chemotherapeutic outflow [18,19]. Interestingly, NO offers unique advantages with respect to conventional drugs such as the absence of MDR, a multitarget action due to its capability to react with all biological components, and a short lifetime (ca. 5 s in tissues), allowing to confine its action to a tightly defined region with a consequent reduction of systemic effects. However, it needs to be considered that the biological effects of NO are strictly dependent on its doses [22,23,24]. While concentrations in the μM range promote apoptosis, cytotoxic action or chemosensitization, concentrations in the pM–nM range encourage tumor progression [25,26]. Therefore, NO donors with precise spatiotemporal control of NO release are highly desirable to realize the full potential of NO [27,28]. To this end, light-activatable NO donors, namely NO photodonors (NOPD), are very appealing due to the superb spatiotemporal control light trigger offers [29,30,31,32,33]. Only in recent years the combination of NOPD with conventional chemotherapeutics has been reported [34,35,36,37], highlighting the double role of photo-released NO as a cytotoxic agent [34] and inhibitor of the cellular efflux pumps [35,36]. Recently, the potential role of NO in combination with protein kinase inhibitors has been proposed, opening new prospects for improving cancer treatments with this class of drugs [38].

**Scheme 1:**
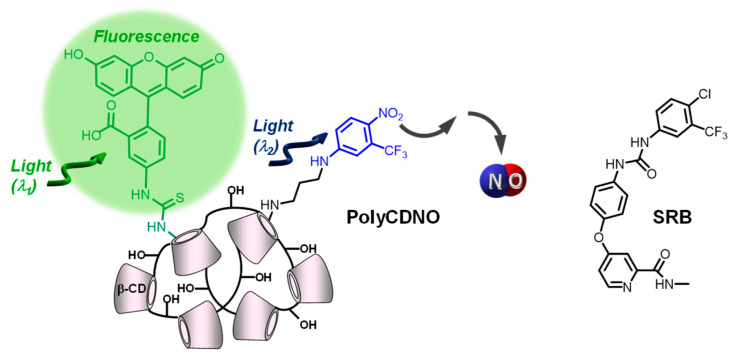
Molecular structures of the β-CD branched polymer PolyCDNO and of SRB.

The above-described increasing development of polymeric systems as a nanocarrier for SRB and the potential of NO in combination with protein kinase inhibitors have stimulated our interest in exploring the capability of our recently developed polymer PolyCDNO (Scheme 1) [39] as a suitable carrier system to enhance SRB anticancer activity.

PolyCDNO is a water-soluble branched β-cyclodextrin (β-CD) polymer covalently integrating a NOPD and a fluorescein isothiocyanate (FITC) moiety within its macromolecular scaffold. β-cyclodextrin branched polymers represent a class of nanocarriers very suitable for SRB encapsulation [40] and have been extensively used in our group to integrate photoactivatable small molecules and deliver them into cancer cells [41,42,43,44,45]. In the case of PolyCDNO, the NOPD and FITC chromophores can be operated in parallel upon excitation with visible light of different wavelengths, resulting in regulated NO release for therapy and green fluorescence emission for imaging [39]. Herein, we explore the suitability of PolyCDNO as a carrier for SRB, investigating the physicochemical and photophysical and photochemical properties of the resulting supramolecular complex and its biological activity against different cancer cell lines.

## 2. Results and Discussion

**Figure 1:**
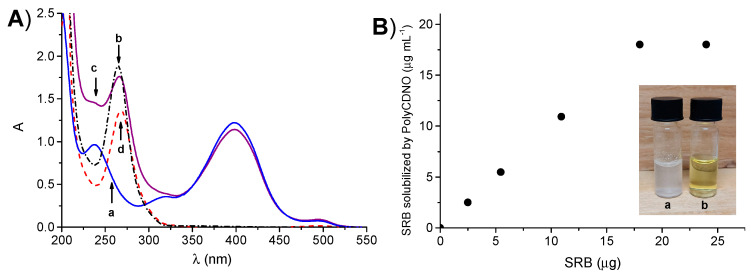
(**A**) Absorption spectrum of an aqueous solution of PolyCDNO (1 mg mL^−1^) (a), a methanol solution of SRB (18 µg mL^−1^) (b) and an aqueous solution of PolyCDNO in the presence of SRB at 18 µg mL^−1^ (c) and the difference spectra c–a (d). (**B**) The amount of SRB solubilized by an aqueous solution of PolyCDNO (1 mg mL^−1^) as a function of the initial amount of SRB. The images in the inset show samples of SRB (18 µg mL^−1^) in the absence (a) and in the presence (b) of PolyCDNO (1 mg mL^−1^) in aqueous medium.

PolyCDNO (MW ca. 420 kD) contains ca. 2.4 % (*w*/*w*) of NOPD and 0.05 % (*w*/*w*) of FITC and is well-soluble in aqueous media. The spectral features of the polymer are dominated by the absorption band of NOPD in the blue region with a maximum at ca. 400 nm and a weak absorption in the green region with a maximum at ca. 500 nm due to the FITC chromophore (a in Figure 1A). The absorption spectrum of SRB in methanol (b in Figure 1A) shows the main absorption band with a maximum at 265 nm, which falls in a wavelength range where the absorption of PolyCDNO is low. These spectral conditions are ideal to study the encapsulation of SRB in the polymer by UV–Vis absorption spectroscopy.

Solubility studies were performed by using a concentration of the polymer of 1 mg mL^−1^. Thin films of SRB, obtained by drying a methanol solution of the drug at different concentrations, were stirred with an aqueous solution of PolyCDNO, and the absorption spectra were then recorded. As shown in Figure 1A (spectrum c), the absorbance values at 265 nm increased as a result of the formation of a host–guest PolyCDNO/SRB supramolecular complex reaching a limiting value of 18 μg mL^−1^ (Figure 1B). Note that the absorption bands of the NOPD and FITC were only slightly affected upon SRB encapsulation, suggesting a negligible interaction between the drug and both these chromophoric units in the ground state. On the other hand, a slight red-shift of the absorption maximum of SRB compared to that in methanol was observed, in agreement with the localization of the drug in an environment of different polarity (spectrum d). The solubilization of SRB can be also noted with the naked eye, as confirmed by the clear solution obtained in the presence of PolyCDNO in contrast to the suspension observed in its absence (a and b in Figure 1B). The complex was stable for several weeks, as demonstrated by the unaltered position and intensity of the absorption spectrum.

The extent of SRB complexation with the polymer in different media was assessed by evaluating the amount of free SRB in the equilibrium with the complex. To this purpose, we centrifuged the PolyCDNO/SRB sample through a polyethersulfone membrane with a MWCO of 5000 Da, allowing the exclusive permeation of free SRB, and analyzed the permeate by HPLC. Independently of the initial SRB content in the PolyCDNO/SRB sample (1.5 and 18 μg mL^−1^), an amount as high as ca. 80% did not permeate through the membrane due to complexation with PolyCDNO, demonstrating that, in the adopted conditions, SRB is in a prevalent complexed form. The permeated amount is ca 0.3 μg/mL, which is reasonable if considering that SRB is practically insoluble in water. The amount of complexed SRB was basically the same also in the cell culture medium, suggesting that the presence of amino acids, vitamins, inorganic salts and glucose does not alter the extent of the complexation in the conditions adopted for the cell studies (vide infra).

**Figure 2:**
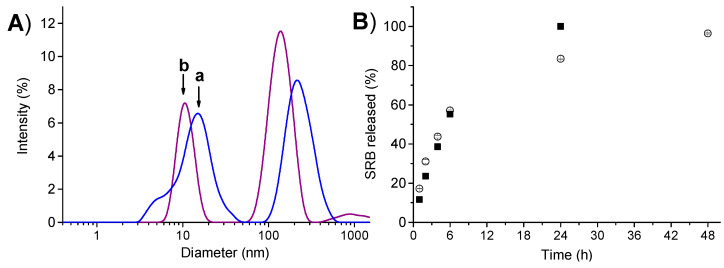
(**A**) Size distribution of PolyCDNO (1 mg mL^−1^) in the absence (a) and in the presence (b) of SRB (18 µg mL^−1^). (**B**) Release profile of SRB (18 μg mL^−1^) free (■) and complexed with PolyCDNO (1 mg mL^−1^) (◯) assessed by dialysis in PBS at pH 7.4 and 37 °C. Data are reported as the mean ± SD (*n* = 3). SD were always lower than 1.65%.

The dynamic light scattering analysis showed that, similarly to the free polymer, the complex PolyCDNO/SRB is polydispersed evidencing two different populations of ca. 10 and 110 nm, respectively (Figure 2A). The slight shift of the size to lower mean values in the case of a complex is indicative of a rearrangement of the polymer network due to noncovalent interactions with SRB. It is worth nothing that the polydispersity of PolyCDNO can be ascribed to the synthetic conditions adopted. We preliminary verified that the large population was not due to the aggregation of the small one, since the addition of ethanol, which should discourage the formation of host–guest interactions between the nitroaniline moiety and CD cavity, did not change the size trend. A possible hypothesis is that the higher solubility of β-CD as compared with that of β-CD-NOPD monomers in the polymerization solvent, results in an initial β-CD cross-linking and, following β-CD-NOPD polymerization, gives rise to two distinct populations.

The release of SRB from the polymer was evaluated by dialysis in a physiological medium (PBS, pH 7.4) in sink conditions. As shown in Figure 2B, the SRB release rate from the complex was lower than that of free drug under the same concentration due to complex formation in the dialysis tube (the polymer could not cross the membrane) and was complete after 2 days.

One of the main requisites for a successful photochemotherapeutic combination is the preservation of the properties of the single functional components after their confinement in a restricted space such as the nanocarrier. This is not trivial due to potential intermolecular processes occurring upon light excitation, which can influence the efficiency and nature of the desired photoinduced processes, leading, in principle, to unexpected photodecomposition pathways. In the present case, the absorption of SRB falls at energy higher than that of NOPD and FITC. This makes any photoinduced energy transfer from the chromogenic units of the polymer to the drug thermodynamically unfeasible. On the other hand, photoinduced electron transfer involving the same components cannot be ruled out. Therefore, the photochemical and photophysical properties of the complex were then investigated and compared with the free polymer.

**Figure 3:**
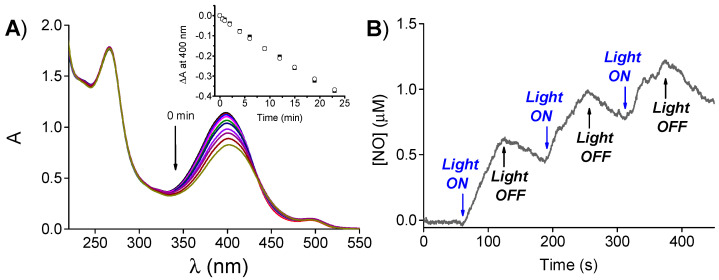
(**A**) Absorption spectral changes observed upon 405-nm light irradiation of an aqueous solution of PolyCDNO (1 mg mL^−1^) encapsulating SRB (18 μg mL^−1^). The inset shows the absorbance changes at 400 nm of PolyCDNO in the presence (◯) and, for comparison, in the absence (■) of SRB. (**B**) NO release profile observed for PolyCDNO (1 mg mL^−1^) in the presence of SRB (18 μg mL^−1^).

Figure 3A shows that irradiation of the complex with blue light leads to the bleaching of the main absorption band of the NOPD at 400 nm. In contrast, no relevant spectral changes are observed either in the region of the FITC at 500 nm or in that of SRB at 265 nm, excluding the involvement of such functional units in any intermolecular photoinduced decomposition process. Accordingly, the rate of the photobleaching was basically the same to that observed for the free polymer (inset Figure 3A).

**Figure 4:**
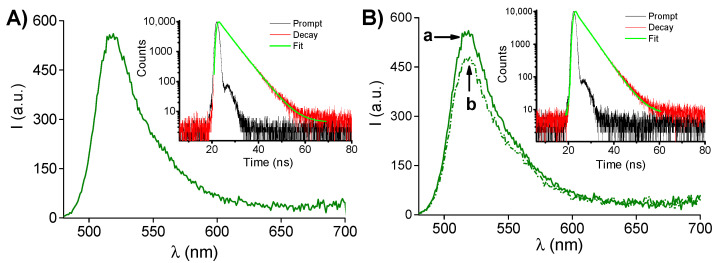
(**A**) Fluorescence emission spectrum (λ_exc_ = 470 nm) of an aqueous solution of PolyCDNO (1 mg mL^−1^) in the presence of SRB (18 μg mL^−1^). The inset shows the fluorescence decay profile monitored at λ_em_ = 530 nm. (**B**) Fluorescence emission spectrum (λ_exc_ = 470 nm) of an aqueous solution of PolyCDNO (1 mg mL^−1^) in the presence of SRB (18 μg mL^−1^) before (a) and after (b) 50 min of light irradiation at 405 nm. The inset shows the fluorescence decay profile monitored at λ_em_ = 530 nm after 50 min of irradiation.

Photo-regulated NO release from the PolyCDNO/SRB complex was demonstrated by direct detection by using amperometric techniques. As illustrated in Figure 3B, NO photorelease from the complex occurred only upon light stimuli with a quantum efficiency Φ_NO_ = 0.006 ± 0.001, very similar to that previously reported for the free polymer (Φ_NO_ = 0.005 ± 0.001) [39] and in excellent agreement with the photobleaching experiments of Figure 4A.

The presence of the FITC in the structure of PolyCDNO is fundamental to track the polymer in a cellular environment. SRB had also no influence on the emissive properties of the polymeric host. In fact, both the static and dynamic features of a typical green fluorescence emission arising from the FITC moiety (Figure 4A and related inset) remained basically unaffected in the presence of SRB, as confirmed by the value of the fluorescence Φ_f_ = 0.51 and a fluorescence decay exhibiting a dominant component (ca. 80%) with a lifetime τ = 4.3 ns, basically the same to those observed for the free polymer [39]. Moreover, these emissive properties remained only slightly affected even after prolonged irradiation times (Figure 4B), confirming the absence of undesired intermolecular processes between the FITC and the byproduct formed after the loss of NO from the NOPD unit.

**Figure 5:**
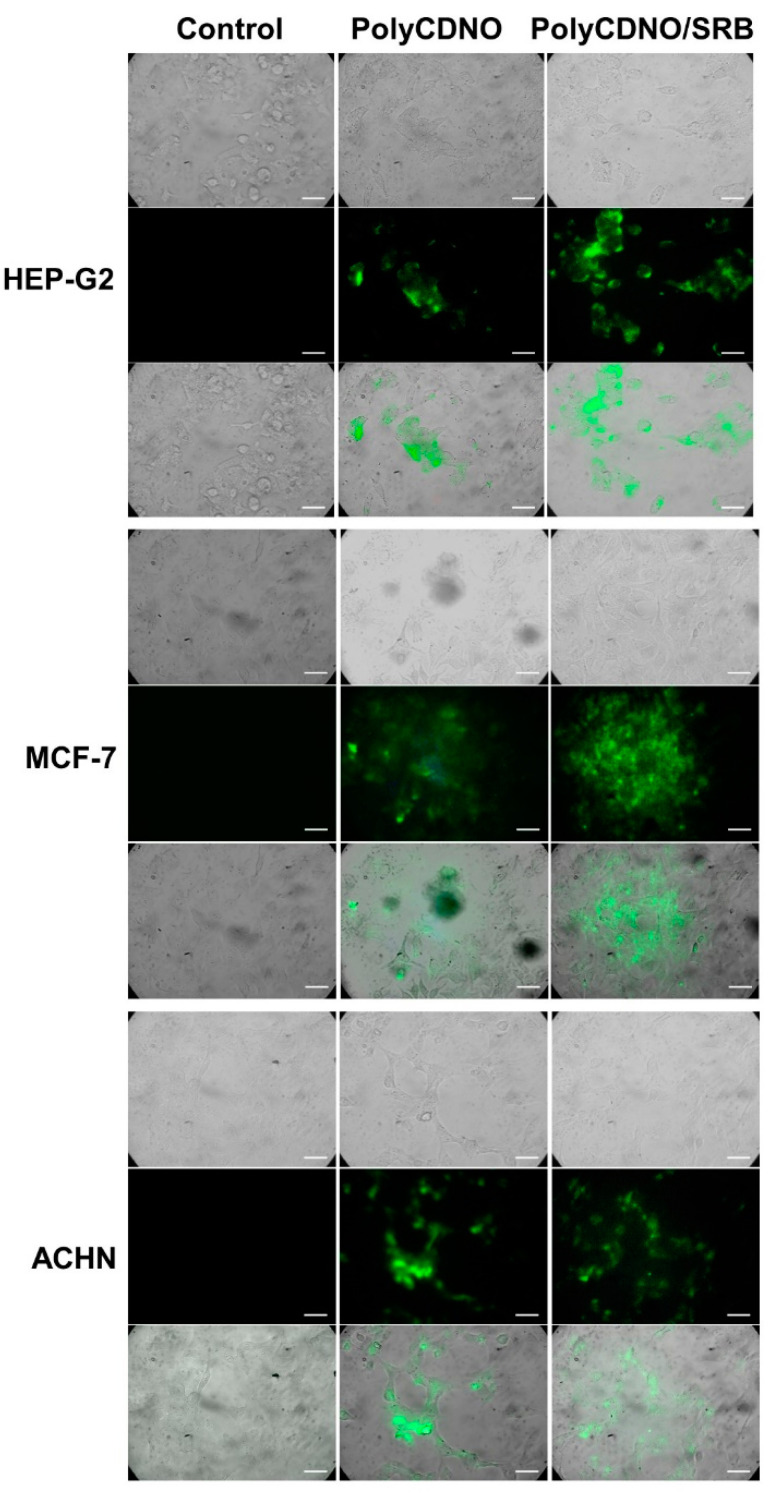
Representative transmission microscopy images (**upper panels**), fluorescence microscopy images collected in the range 500–580 nm and observed upon excitation at λ_exc_ = 488 nm (**central panels**) and merged images (**lower panels**) of different cancer cells incubated for 4 h under different conditions. PolyCDNO (1 mg mL^−1^ SRB (1.5 µg mL^−1^). Scale bar = 50 µm.

The preservation of the emissive properties of the FITC label after complexation of PolyCDNO with SRB and the fact that the complexation capability of PolyCDNO was not affected by the cell culture medium allowed us to explore the cellular uptake of the PolyCDNO/SRB complex by fluorescence microscopy. Figure 5 shows representative fluorescence images of HEP-G2 hepatocarcinoma, MCF-7 breast cancer and ACHN kidney adenocarcinoma cells obtained after 4 h of incubation with the complex and, for comparison, with the free polymer in FBS-free media excited at 488 nm and monitored in the green channel (500–580 nm). It is evident that, in all cases, the typical green fluorescence originating from the FITC tag arises from the cell cytoplasm, where both the complexed and the free polymer are present in the form of brighter aggregates.

**Figure 6:**
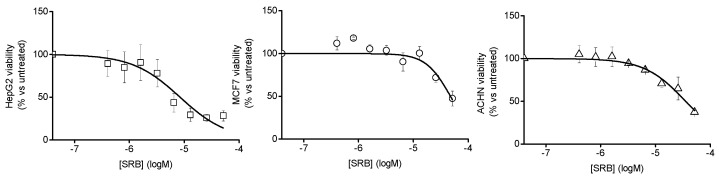
Viability of different cancer cell lines as a function of the concentration of free SRB.

Cell mortality experiments were carried out with all the above cell lines. Preliminarily, the cell viability was investigated as a function of the concentration of the free SRB in order to determine the IC_50_ values. These resulted in 3.8 µg mL^−1^, 22.0 µg mL^−1^ and 16.3 µg mL^−1^ in the case of HEP-G2, MCF-7 and ACHN cancer cells, respectively (Figure 6). Additionally, our recent work showed that free PolyCDNO induces significant cell mortality due to the cytotoxic action of the NO generated only for times longer than 40 min [39].

**Figure 7:**
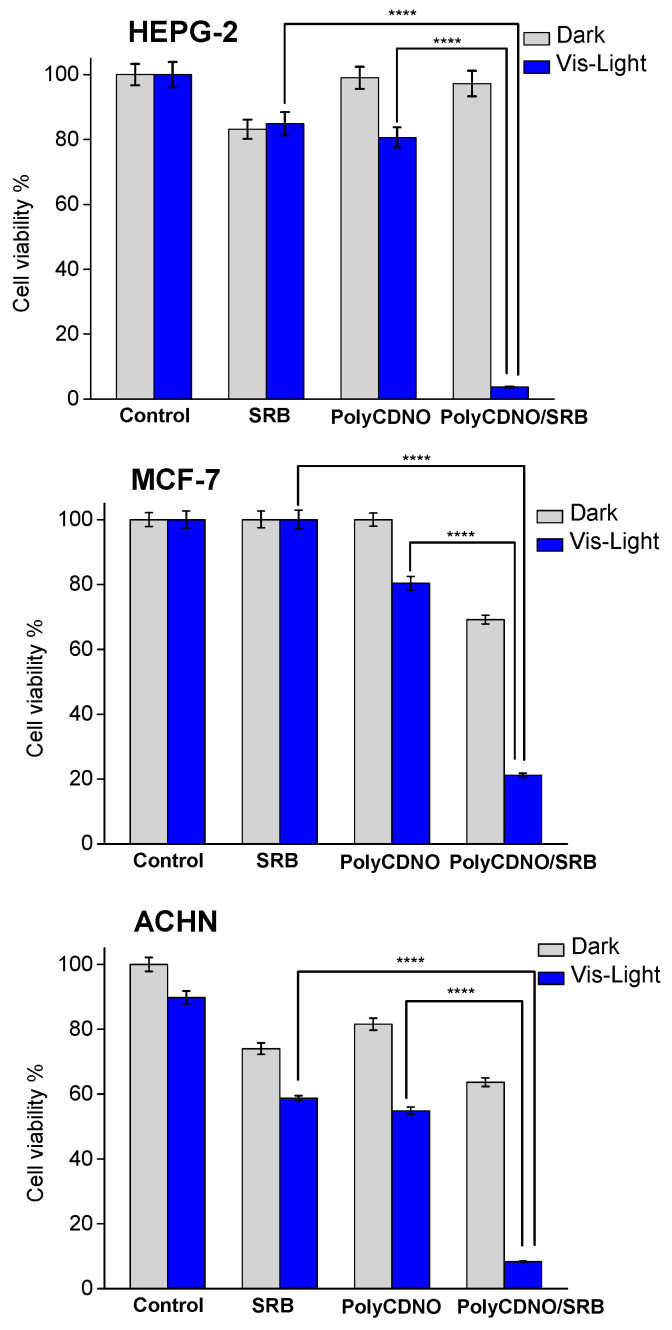
Cell viability observed 24 h after incubating different cancer cells for 4 h with free SRB (1.5 µg mL^−1^), PolyCDNO (1 mg mL^−1^) and the complex PolyCDNO/SRB in the dark and upon 40 min of irradiation at λ_exc_ > 400 nm. (**** *p* < 0.0001; *n* = 3).

On the basis of these findings, we chose to test the activity of the PolyCDNO/SRB under visible light excitation and, for comparison, in the dark using a concentration of the drug of 1.5 µg mL^−1^, which was well below the IC_50_ for all the cell lines tested, and an irradiation time of 40 min, which allowed to generate a moderately cytotoxic NO dose. The overall results are reported in Figure 7.

According to the chosen experimental conditions, free SRB exhibited either negligible or moderate toxicity against all the cell lines in the dark. A slight increase of toxicity was observed only in the case of ACHN cells. This photoinduced effect was probably due to a specific sensitivity of this cell line to irradiation light sources, as demonstrated by the comparable lowering of cell viability noted also in the control sample and according to the inability of SRB to absorb photons in the visible range. Analogously, PolyCDNO was well-tolerated in the dark and exhibited a slight increase of toxicity upon light irradiation according to the production of moderate NO doses. Interestingly, a very remarkable reduction of cell viability was observed in the case of the complex in all the cancer cell lines, with the effect being more pronounced in the case of HEP-G2 hepatocarcinoma cells. The potentiation indexes (%viability SRB/%viability polyCDNO/SRB) were, in fact, ca. 28, 5 and 8 for the HEP-G2, MCF-7 and ACHN cancer cell lines, respectively. Note that the photochemical experiments have demonstrated that NO photorelease efficiency is not affected by the presence of SRB (see Figure 4B) and that photoexcitation of the complex does not lead to any photodecomposition of SRB (see Figure 4A). Therefore, such a dramatic enhancement of cell mortality cannot be ascribed to either an increase of the NO photorelease efficiency or an involvement of a potentially toxic new photoproduct in the case of the complex. As a consequence, it appears clear the occurrence of a notable synergistic action between the chemotherapeutic and the NO, individually used at not significantly toxic doses, when they are combined together in the supramolecular complex. Since it has been demonstrated that SRB induces the generation of ROS in human HCC cell lines in vivo and in vitro in a dose-dependent manner [46], we hypothesize that ROS, especially superoxide anion, reacts with NO, forming the highly cytotoxic peroxynitrite through a well-known diffusion-controlled process [47]. Biological studies addressed to gain insights into the mechanisms at the basis of such synergistic actions deserve the forthcoming attention.

## 3. Materials and Methods

### 3.1. Materials

All chemicals were purchased by Sigma-Aldrich, (Milan, Italy), and used as received. PolyCDNO was synthesized in one step starting from the copolymerization of native β-CD and the monomeric β-CD derivatives incorporating FITC and NOPD, respectively, with epichlorohydrin as the cross-linking agent, according to our previously reported procedure [39]. The SRB concentration was determined by absorption spectroscopy, using a molar extinction coefficient of 41,280 M^−1^ cm^−1^ at 265 nm in the MeOH solution and 32,500 M^−1^ cm^−1^ at 268 nm when complexed within PolyCDNO. All solvents used for the spectrophotometric studies were spectrophotometric grade. Milli-Q water was used for the polymer solubilization for the chemical and photochemical assays.

### 3.2. Instrumentation

UV–Vis spectra absorption and fluorescence emission spectra were recorded with a JascoV-560 spectrophotometer and a Spex Fluorolog-2 (mod. F-111) spectrofluorometer, respectively, in air-equilibrated solutions using quartz cells with a path length of 1 cm. Fluorescence lifetimes were recorded with the same fluorimeter equipped with a TCSPC Triple Illuminator. The samples were irradiated by a pulsed diode excitation source (Nanoled) at 455 nm, and the decay was monitored at 530 nm. The system allowed measurement of the fluorescence lifetimes from 200 ps. The multiexponential fit of the fluorescence decay was obtained using the following equation:

I(*t*) = Σα_i_exp(^−*t*/τi^) (1)

Direct monitoring of NO release in the solution was performed by amperometric detection (World Precision Instruments), with an ISO-NO meter, equipped with a data acquisition system and based on the direct amperometric detection of NO with a short response time (<5 s) and sensitivity range 1 nM–20 µM. The analog signal was digitalized with a four-channel recording system and transferred to a PC. The sensor was accurately calibrated by mixing standard solutions of NaNO_2_ with 0.1-M H_2_SO_4_ and 0.1-M KI according to the reaction:

4H^+^ + 2I^−^ + 2NO_2_^−^ → 2H_2_O + 2NO + I_2_ (2)

Irradiation was performed in a thermostated quartz cell (1-cm path length and 3-mL capacity) using the continuum laser with λ_exc_ = 405 nm. The NO measurements were carried out under stirring with the electrode positioned outside the light path in order to avoid NO signal artefacts due to photoelectric interference on the ISO-NO electrode.

The size distribution was determined on a Zetasizer Nano ZS (Malvern Instruments Ltd., Malvern, UK).

### 3.3. NO Photorelease and Fluorescence Quantum Yields

The NO photorelease quantum yield, Φ_NO,_ was determined at λ_exc_ = 405 nm within the 20% transformation of PolyCDNO by using the following equation:

Φ_NO_ = [PolyCDNO] × *V*/*t* × (1 − 10^−A^) × I (3)

where [PolyCDNO] is the concentration of phototransformed 1, *V* is the volume of the sample, *t* is the irradiation time, A is the absorbance of the sample at the excitation wavelength and I the intensity of the excitation light source. The concentration of the phototransformed 1 was determined spectrophotometrically by taking into account the absorption changes at 400 nm and a Δε_400_ = 10,000 M^−1^ cm^−1^. I was calculated by potassium ferrioxalate actinometry.

The fluorescence quantum yield Φ_f_ was determined using optically matched solutions at the excitation wavelength of PolyCDNO, used as a secondary standard, and the complete absorbance at the excitation wavelength was less than 0.1 in all cases.

### 3.4. Preparation of the PolyCDNO/SRB Complex

A stock solution of SRB in MeOH of 38 µM was utilized, and the solvent was evaporated under reduced pressure at 25 °C. The resulting film was rehydrated with an aqueous solution of PolyCDNO (1 mg mL^−1^) by stirring for 12 h at room temperature.

For the biological assays and some of the chemical evaluations, the complexes were prepared by rehydrating the SRB film with a solution of PolyCDNO (1 mg mL^−1^) in DMEM high glucose (without phenol red).

### 3.5. Extent of Complexation of SRB in PolyCDNO/SRB and SRB Release

The amount of SRB complexed within PolyCDNO was assessed by centrifugation of a PolyCDNO/SRB sample (18 μg/mL) at 9000× *g* for 20 min through VIVASPIN^®^ 6 (MW 5 kDa). The same experiment was carried out also on PolyCDNO/SRB prepared analogously in DMEM high glucose (without phenol red). The SRB content in the eluate was analyzed by HPLC, as described below.

The PolyCDNO/SRB complex prepared in DMEM (0.5 mL) was placed in a dialysis bag (SpectraPor 3500 da) and poured in 5 mL of Phosphate-Buffered Saline (PBS; 0.01-M phosphate buffer, 0.0027-M potassium chloride and 0.137-M sodium chloride) at pH 7.4 and 37 °C under stirring. At different time points, 1 mL was collected and replaced with fresh medium. The samples were analyzed by HPLC as described below to evaluate the released SRB. As a control, free SRB solubilized in methanol and added in the dialysis bag at the same concentration as the complex was dialyzed in similar experimental conditions.

The quantitation of SRB was performed by High-Performance Liquid chromatography on a Shimadzu apparatus equipped with an LC-10ADvp pump, an SPD-10Avp ultraviolet–visible (UV–Vis) detector and a C-R6 integrator. A SphereClone ODS 25-μm C18 column (250 × 4.6 mm, 80 Å) was used (Phenomenex, Torrance, CA, USA). The mobile phase was water containing 2% triethylamine adjusted to pH 5.4 and acetonitrile with phosphoric acid at 65:35 (*v*/*v*) pumped at a 1-mL/min flow rate. The UV detector was set at 265 nm. A calibration curve of SRB in methanol was constructed in the concentration range 0.2–100 μg/mL (r^2^ = 0.9, LOQ 0.1 μg/mL).

### 3.6. Biological Assays

#### 3.6.1. Cell Lines

The in vitro studies were carried out in a panel of three cancer cell lines: HepG2, ACHN and MCF-7 (all from American Type Culture Collection, ATCC, Manassas, VA, USA). The cell lines were chosen according to the SRB clinical applications. The cell lines were maintained in Dulbecco’s modified Eagle’s medium (DMEM, Sigma-Aldrich, Milan, Italy) supplemented with 10% fetal bovine serum and 1% penicillin/streptomycin. Cells were cultivated in 75-cm^2^ culture flasks (EuroClone, Milan, Italy) at 37 °C in a humidified atmosphere of 95% air, 5% CO_2_. The trypsin-EDTA solution (Sigma-Aldrich, Milan, Italy) was used to detach cells from the bottom of the flask.

#### 3.6.2. Fluorescence Microscopy

Cells were cultured in glass coverslips inserted into 24- or 6-well culture dishes. The medium was removed and replaced with either the free PolyCDNO or its complex with SRB for 4 h. Then, the cells were first washed with PBS, then fixed with 4% formaldehyde. The images were acquired by a microscope (Zeiss Axioplan, Carl Zeiss, Milan, Italy) equipped with a camera (Axicam, Carl Zeiss, Milan, Italy).

#### 3.6.3. Viability Assay

The cells were seeded as previously described, and after 4 h of incubation with free SRB or SRB loaded into PolyCDNO, they were washed once with PBS and irradiated in PBS with a 150-W Xe lamp through a cut-off filter at λ = 400 nm. Immediately after irradiation, the PBS was replaced with the complete medium, and the cell plates were brought back to the incubator. Cell viability was measured with the MTT test after an additional 24 h. The results were reported as the percentage of viability versus untreated controls. The free SRB was dissolved in 100% DMSO (Sigma-Aldrich, Milan, Italy) and diluted with serum-free medium to the desired concentration, with a final DMSO concentration of 0.1%. The control cultures received equal amounts of DMSO as the solvent control.

The cytotoxic effect in the dark was measured with the MTT (3-(4,5-dimethyl-2-thiazolyl)-2,5-diphenyl-2H-tetrazolium bromide) assay. Cells (1 × 10^4^) were seeded in 96-well flat bottom plates and incubated for 48 h until the monolayer reached a confluency of approximately 80% and then incubated with a scalar concentration of SRB diluted in serum-free medium for 24 or 4 h. The short-term incubation of 4 h was followed by an additional 24 h of cell growth in drug-free medium with serum (4 h + 24 h). After the indicated time, the medium was replaced with 180 μL of medium and 20 μL of MTT solution (stock solution at 5 mg/mL), and the wells were incubated for 3 h at 37 °C. In this condition, mitochondrial dehydrogenases of viable cells conversed the tetrazolium ring of MTT into formazan crystals that were solubilized in 100 μL of DMSO. The absorbance was measured at 550 nm with a Varioskan™ LUX multimode microplate reader (Thermo Fisher Scientific, Milan, Italy). The cytotoxicity index was calculated using the untreated cells as a negative control (100% viability), and the IC_50_ was extrapolated from the dose–response graph by nonlinear regression analysis using GraphPad Prism software v. 7.00 (San Diego, CA, USA).

## 4. Conclusions

Our results demonstrate a viable strategy to enhance the anticancer activity of SRB by using low therapeutic doses thanks to the combination with NO. This is achieved through a biocompatible, water-soluble fluorescent β-cyclodextrin-based polymer able to release NO and emit green fluorescence under the exclusive control of light stimuli. This multifunctional polymer is able to effectively entrap SRB, significantly increasing its solubility in aqueous medium, retaining the photochemical properties after the drug encapsulation and releasing SRB in an aqueous medium. The host–guest supramolecular complex internalizes in different cancer cell lines, where it can be visualized thanks to its fluorescent properties, localizing mainly at the cytoplasmic level. Toxicity experiments carried out at the SRB concentration well below its IC_50_ and regulating the amount of NO released through appropriate light tuning clearly point out a remarkable synergistic action between NO and SRB, which results in a high level of cell mortality.

To our knowledge, this is the first example showing the benefit of a nanoplatform combining a multi-kinase inhibitor such as SRB with a light-regulated NO release. In view of the increasing interest in combining NO with protein kinase inhibitors [22], and considering the very critical dependence of the biological effects of NO by its concentration [23,24,25,26,27], these findings may open up interesting avenues not only to potentiate the anticancer action of SRB but, in principle, to reduce its side effects given the low doses used.

## Data Availability

Not applicable.

## Sample Availability

Samples of the compounds are not available from the authors.
